# Nasal allergen challenge with dissolved birch tree pollen tablets

**DOI:** 10.1038/s41598-025-27356-4

**Published:** 2025-11-10

**Authors:** Eirini Paziou, Karin Jamil Ali, Susanna Kumlien Georén, Agneta Karlsson, Emma Nilsson, Laila Hellkvist, Lars Olaf Cardell

**Affiliations:** 1 Department of ENT Diseases, Karolinska University Hospital, Stockholm, Sweden; 2 Division of ENT Diseases, Department of Clinical Sciences, Intervention and Technology, Karolinska Institutet, Stockholm, Sweden

**Keywords:** Allergy, Pollen, Rhinitis, Diagnosis, Challenge tests, Birch, Diseases, Drug discovery, Health care, Medical research

## Abstract

The nasal allergen challenge (NAC) is a valuable diagnostic tool for both clinicians and researchers in the field of allergy and its management. In Europe, an aqueous extract Aquagen SQ (ALK-Abelló) was historically the most widely used solution for this purpose. Its production ceased in 2019, creating a gap in available resources. This clinical trial evaluated the feasibility of using dissolved birch pollen tablets, ITULAZAX 12-SQ Bet (ALK-Abelló) tablets as an alternative for NAC. A total of 69 participants with confirmed birch pollen allergy and 20 healthy controls underwent NAC using either dissolved ITULAZAX, dissolved placebo tablets, or a 0.9% saline solution. The primary objective was to assess the sensitivity of NAC with dissolved ITULAZAX. The method demonstrated a sensitivity of 96% and a specificity of 95%. These findings support the use of dissolved ITULAZAX as a reliable and accessible allergen source for nasal allergen challenges in both clinical practice and research.

**Supplementary Information:**

The online version contains supplementary material available at 10.1038/s41598-025-27356-4.

## Introduction

Pollinosis, commonly referred to as hay fever, is a seasonal allergic disorder caused by exposure to airborne pollens from trees, grasses, and weeds. The clinical spectrum ranges from nasal and ocular manifestations —such as allergic rhinitis and conjunctivitis—to respiratory symptoms including pollen-induced bronchitis and, in some cases, asthma exacerbations^1^. Less frequently, pollinosis may present with oral allergy syndrome, urticaria, or, in rare cases, angioedema, gastrointestinal symptoms, and even systemic anaphylaxis^2^. Diagnosis relies on a thorough clinical history demonstrating a temporal relationship between symptoms and pollen exposure, and is confirmed by allergen-specific testing, most commonly skin prick testing and/or the detection of serum-specific IgE antibodies.

The nasal allergen challenge (NAC) is a well-established diagnostic procedure for confirming pollen allergy, particularly in cases where discrepancies arise between clinical history and test results^3,4^. In addition to its diagnostic value, NAC has an important role in clinical research, where it is employed to assess the efficacy of allergen immunotherapy (AIT)^5,6^ and to evaluate the therapeutic effectiveness of pharmacological interventions such as antihistamines and intranasal corticosteroids^7–9^. Importantly, NAC is considered very safe in both pediatric and adult patients with rhinitis, with or without concomitant asthma, as studies assessing lower airway responses via spirometry have demonstrated no significant decline in FEV1^10,11^.

This test elicits a response from the nasal mucosa by controlled exposure to allergens. For many years, Aquagen (ALK-Abelló) has been the most widely used and well-established preparation for performing NAC in patients with pollen allergies in most parts of Europe. However, production of this product ceased in 2019, leaving clinicians and researchers in search of suitable alternatives.

A novel approach utilizing dissolved lyophilized tablets, originally developed for sublingual immunotherapy (SLIT), has been introduced^12^. Olivieri et al.^12^ investigated the minimum concentration of dissolved lyophilized tablets required to elicit a positive nasal challenge response in a small cohort of patients with birch and/or house dust mite allergies. This method was validated by a recent study which demonstrated that dissolved GRAZAX 75,000 SQ-T (ALK-Abelló) yielded results comparable to traditional methods in patients with grass allergies^13^. Clinical efficacy and tolerability for allergen specific immunotherapy (AIT) with Birch lyophilizate tablet ITULAZAX 12-SQ bet (ALK- Abelló) is well established^14^. Therefore, it seems reasonable to assume that the same method could be applied to dissolved ITULAZAX.

This clinical trial aims to assess the feasibility of conducting nasal allergen challenge (NAC) using dissolved ITULAZAX tablets. The present trial design including the trial population has been selected based on the recommendation from the European Medicines Agency (EMA) Committee for medicinal products for human use (CHMP) guideline on clinical evaluation of diagnostic agents (EMA. Guideline on clinical evaluation of diagnostic agents. CPMP/EWP/1119/98. 2008).

An additional benefit of this proposal is that the allergen composition of the provocation product is the same as the final product which the patients could be treated with. This may enhance patient motivation and adherence to treatment. From a practical perspective, the dissolving process is easier for the physicians compared to the more complex procedure of the previously used Aquagen SQ.

## Methods

This study is a national, single-center trial designed to establish a Nasal Allergen Challenge (NAC) method using dissolved ITULAZAX. The Swedish Ethical Review Authority approved the protocol, Uppsala (Ref. 2021–05597-01), and the Swedish Medical Products Agency (Ref. 5.1–2022.1-73533). In accordance with international trial registration requirements, the study was registered at ClinicalTrials.gov (ID: NCT06085963) on September 27, 2023.

The sample size for this trial is based on empirical considerations, as there are no hypothesis tests where statistical power needs to be ensured. No formal sample size calculations have been performed. The performance of the NAC follows the Position Paper of the EAACI^4^ on the standardization of allergen challenges, and all methods were performed in accordance with the relevant guidelines and regulations.

A total of 69 adults (ages 18–65) with a clear history of birch pollen allergy and 20 non-atopic control participants (ages 18–65) were recruited through advertisements at the ENT department of Karolinska University Hospital in Stockholm, between March 2023 and December 2024. Written, informed consent was obtained from all participants prior to their enrollment.

All patients had normal physical examinations which included anterior rhinoscopy and rigid endoscopy. The control group had a documented negative clinical history for allergic rhinitis, as well as negative results on the Skin Prick Test (SPT) and serum IgE testing for birch, grasses, house dust mites, cat, dog, horse, ragweed, mugwort, and mould. In contrast, the birch allergy group had a documented clinical history of moderate to severe tree pollen allergy according to Allergic Rhinitis and its Impact on Asthma (ARIA) guidelines^15^ for at least two seasons prior to the study, along with positive SPT and serum IgE results for birch. The inclusion and exclusion criteria are outlined in Appendix 1.

The demographic characteristics of the participants are presented in Table 1. It should be noted that the reported symptoms of rhinitis, conjunctivitis, asthma, and eczema reflect patients’ recall of the last birch pollen season, as documented in the case report forms (CRFs) during the screening visit.

**Table 1:**
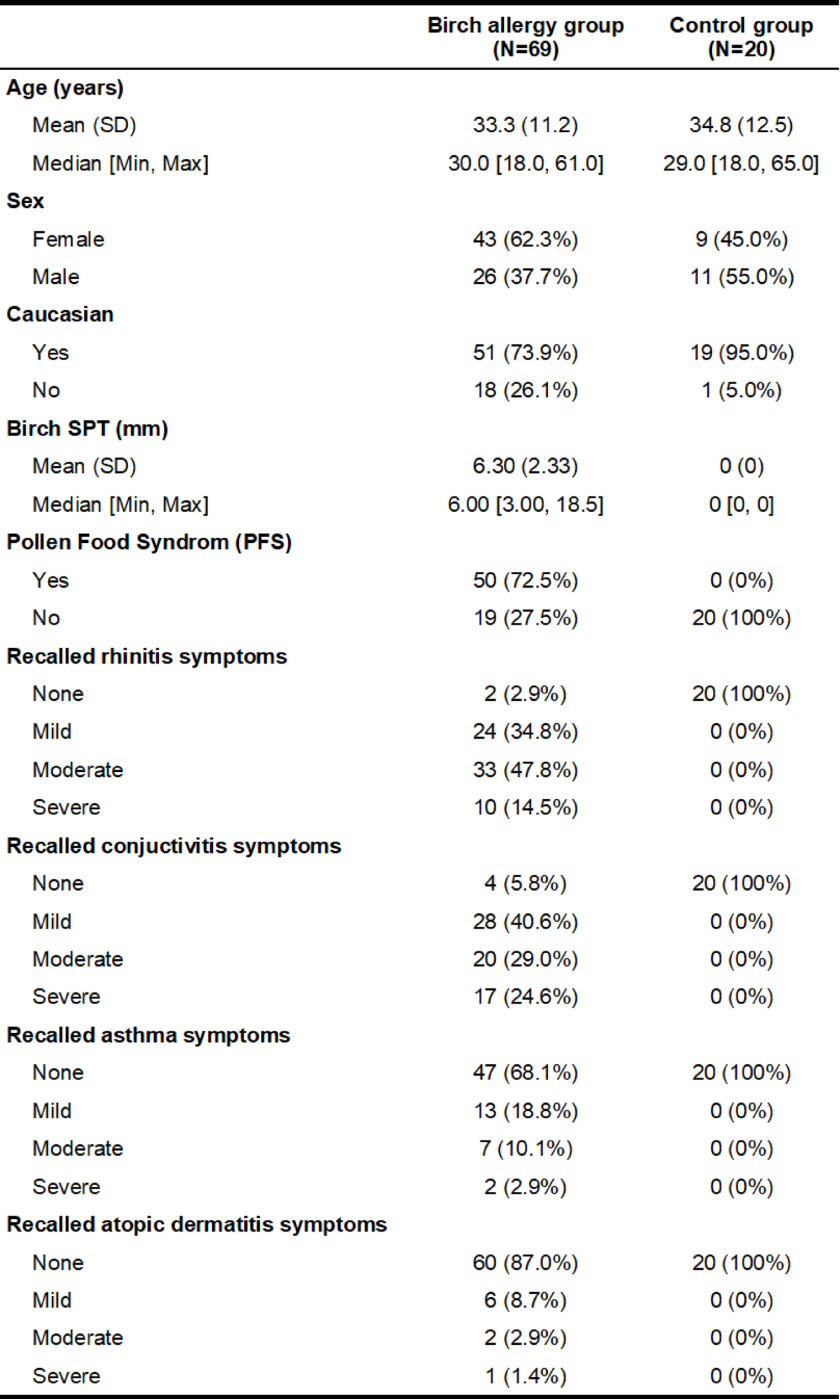
Baseline characteristics.

All challenges were conducted at least four weeks before or after the birch pollen season. NACs were performed using either dissolved ITULAZAX tablets, diluted in 0.9% saline to a concentration of 1 SQ-Bet/ml (with preparation details available in Appendix 2), or a placebo solution, which was prepared identically to the active ITULAZAX stock solution or with 0.9% saline only. Certain medications required a withdrawal period before NACs, as recommended by the EAACI position paper^16^.

All investigational medicinal products (IMPs) were manufactured by ALK. The placebo tablets were designed to closely resemble the active IMP in appearance, smell, and taste. The placebo tablets were labeled in accordance with LVFS 2011:19.

The MAD110 Nasal Device, used for the NAC, delivered 0.1 mL per puff, with one puff administered into each nostril, resulting in an effective dose of 0.2 standardized quantity units (1 SQ-Bet/ml) per nasal cavity (equivalent to 10,000 SQ-U/ml). The rational for using 0.2 SQ-Bet corresponding to 10 000 SQ-U is based on clinical experience with Aquagen SQ, where 10,000 SQ-U has been established as both a safe and effective active dose for nasal provocation. Since Aquagen SQ and ITULAZAX are identical in allergen composition it´s assumable that that the dissolved products at comparable strengths should give similar results.

20 non-atopic controls were challenged with placebo tablet dissolved in 0,9% saline solution and 0,9% saline solution only to ensure negative reactivity to the tablet excipients and the nasal provocation itself. This procedure was conducted with a single-blind, cross-over design, with 10 subjects starting with the placebo tablet dissolved in saline and 10 subjects beginning with saline solution alone. After 30 min, each group received the alternate solution.

For the 69 birch-allergic participants, the NAC began with a 0.9% saline solution to confirm negative reactivity to the challenge as method. After 30 min, the challenge continued with dissolved ITULAZAX.

Total Nasal Symptom Scores (TNSS; Table 2) and peak nasal inspiratory flow (PNIF) were recorded prior to NAC and at 5, 15, and 30 min post-challenge. The challenges were initiated only if the baseline TNSS was 2 or less.

**Table 2:** Total nasal symptom score (TNSS) on a 0–12 likert scale.

Symptom severity	Score (points)
Rhinorrhea (no, mild, moderate, severe)	0–3
Nasal obstruction (no, mild, moderate, severe)	0–3
Sneezing (no, mild, moderate, severe)	0–3
Nasal itching (no, mild, moderate, severe)	0–3

A positive NAC result was defined as follows:

- Significant change in subjective measurement: TNSS increase of ≥ 5 points or.
- Significant change in objective symptoms: PNIF decrease of ≥ 40% or.
- Moderate change in both objective and subjective measures: TNSS increase of ≥ 3 points and PNIF decrease of ≥ 20%.

The primary outcome of this study was to evaluate the sensitivity of NAC with dissolved ITULAZAX. Sensitivity was calculated as the percentage of participants in the birch pollen allergy group who tested positive on the provocation out of the total number of birch pollen allergy participants.

The secondary outcome was to assess the specificity and tolerability of NAC with dissolved ITULAZAX. Specificity was calculated as the percentage of non-allergic control participants who tested negative on the NAC out of the total number of control participants.

## Results

Enrollment commenced in March 2023 and concluded in December 2024, during which a total of 96 participants with suspected birch allergy and 30 non-allergic controls were screened. After applying exclusion criteria, 69 participants remained in the birch allergy group, and 20 participants were retained in the control group. The reasons for exclusion are outlined in Table 3.

**Table 3:**
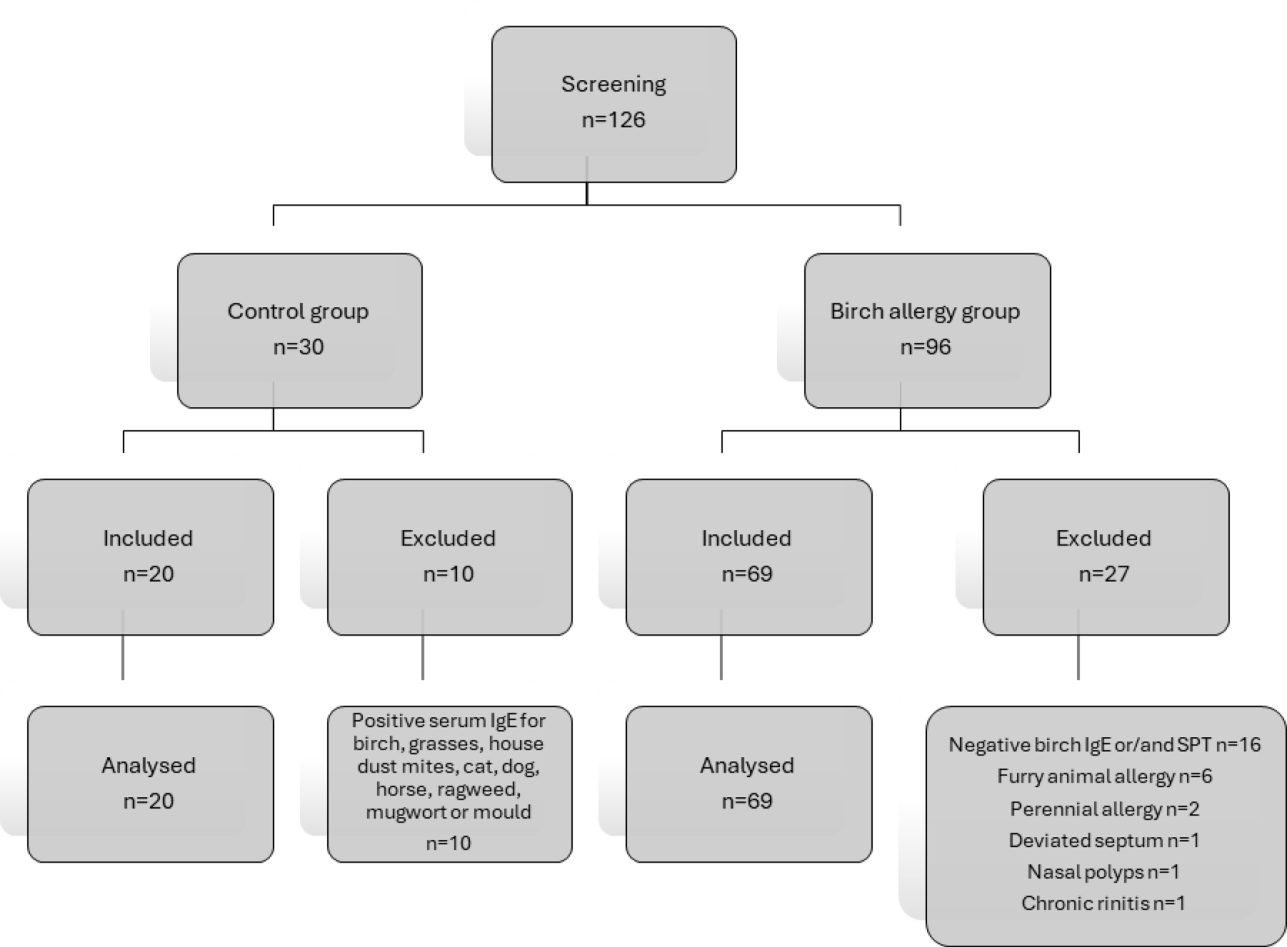
Reasons for exclusion.

All participants completed both challenges.

Ten participants in the control group were initially provoked with a 0.9% saline solution. All challenges were negative. Immediately afterward, they underwent provocation with a placebo tablet dissolved in 0.9% saline solution. All challenges but one remained negative. For this participant, PNIF measurements during the saline challenge were: baseline 90 L/min, 5 min 100 L/min, 15 min 70 L/min, and 30 min 90 L/min. During the placebo challenge, PNIF values were: baseline 150 L/min, 5 min 110 L/min, 15 min 140 L/min, and 30 min 90 L/min. The TNSS remained 0 throughout both challenges. Although a transient decrease in PNIF was observed under the placebo challenge, the overall pattern and lack of subjective symptoms suggest that this participant did not have a true positive response. We report these intermediate PNIF values to maintain full transparency and allow accurate interpretation of the results. The remaining 10 participants in the control group underwent the reverse sequence of challenges, with no positive responses observed. The maximum TNSS observed was 1, and the most significant PNIF reduction was 27% across the challenges. Consequently, NAC in 19/20 healthy controls did not impact TNSS or PNIF (Figue1 and 2). As a result, 19 out of the 20 participants tested negative in both challenges, yielding a specificity of 95% for the method.

**Fig. 1:**
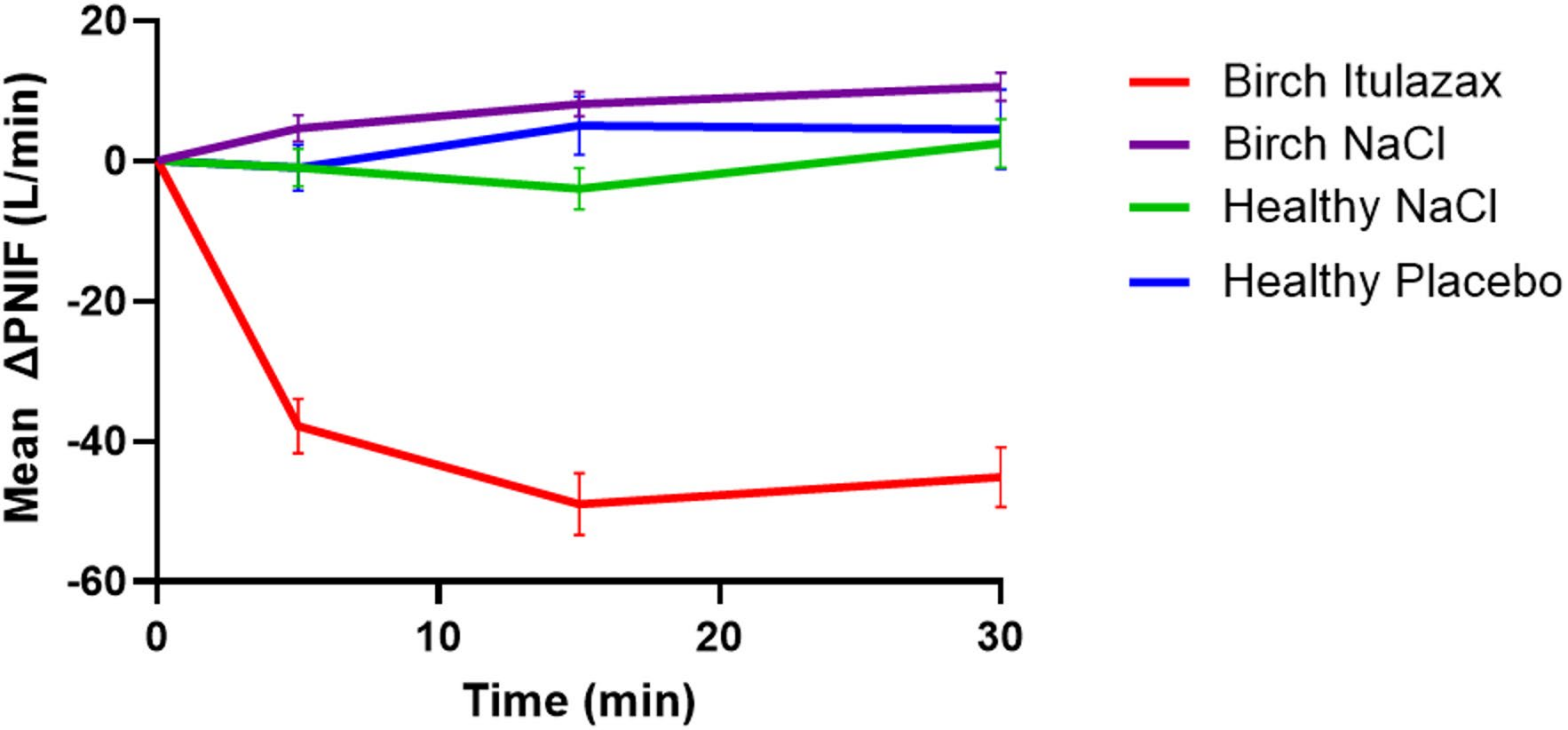
Mean (± SEM) change from baseline PNIF (ΔPNIF), after challenges with 0.9% saline solution (NaCl), placebo, and birch lyophilizate tablets (ITULAZAX).

**Fig. 2:**
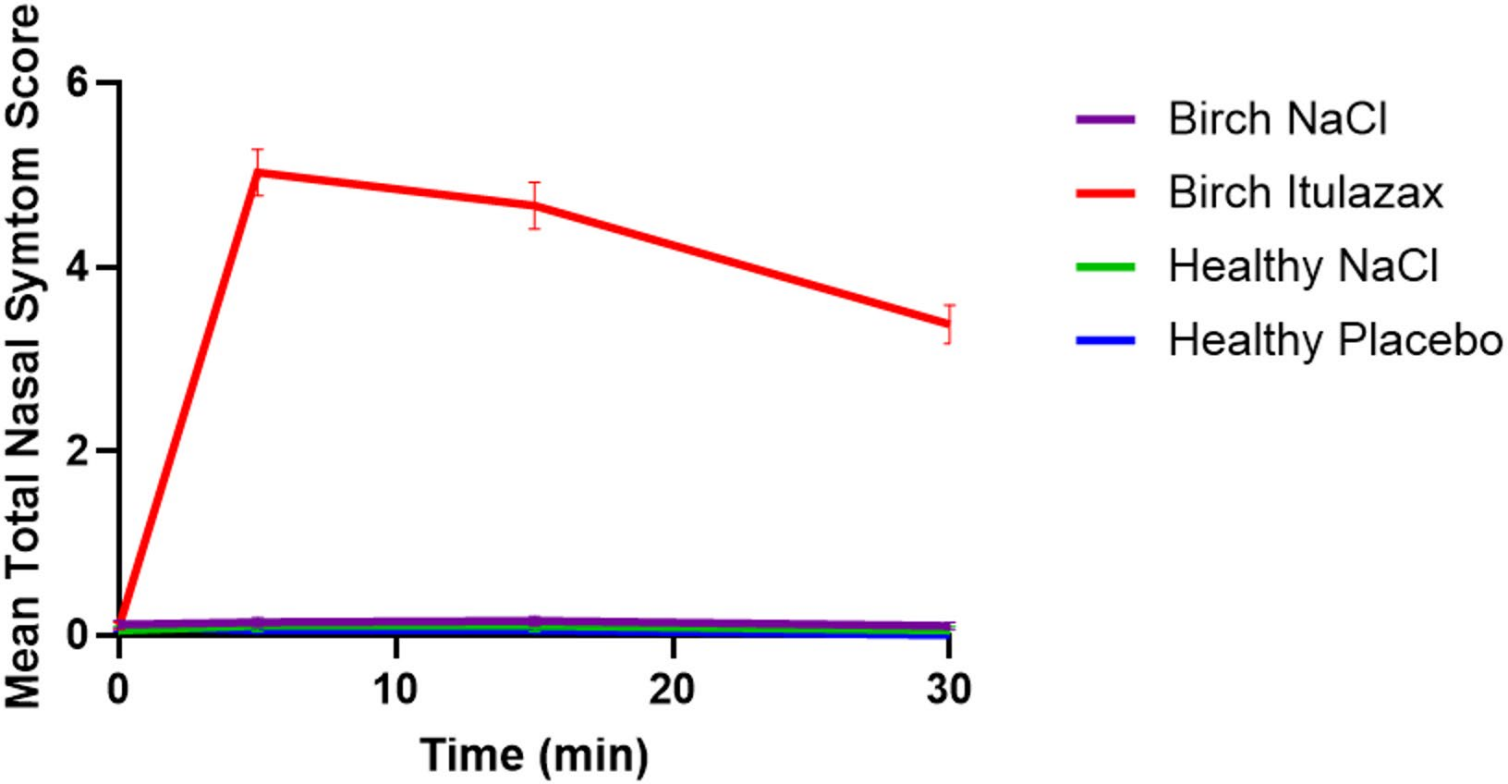
Mean (± SEM) of total nasal symptom scores (TNSS) after challenges with 0.9% saline solution (NaCl), placebo, and birch lyophilizate tablets (ITULAZAX).

The 69 patients in the birch allergy group were first challenged with a 0.9% saline solution, which was negative in all cases. This was followed by nasal provocation with dissolved ITULAZAX. Although two patients mainly suffered from severe conjunctivitis symptoms and reported hardly any rhinitis symptoms during the previous pollen season, their nasal provocations still yielded positive results. Only 3 NACs with dissolved ITULAZAX were negative but very close to the threshold for a positive test result. Among these, two patients experienced an increase from baseline TNSS up to 4 points, with only a 10% decrease from baseline PNIF, while one patient had a 36% decrease in PNIF and a 2-point increase in TNSS. Peak TNSS was observed at 5 min, while the lowest PNIF occurred at 15 min (Figs. 1 and 2). To conclude, 66 of the 69 patients had a positive NAC with dissolved ITULAZAX, resulting in a 96% sensitivity for this method.

**Fig. 3:**
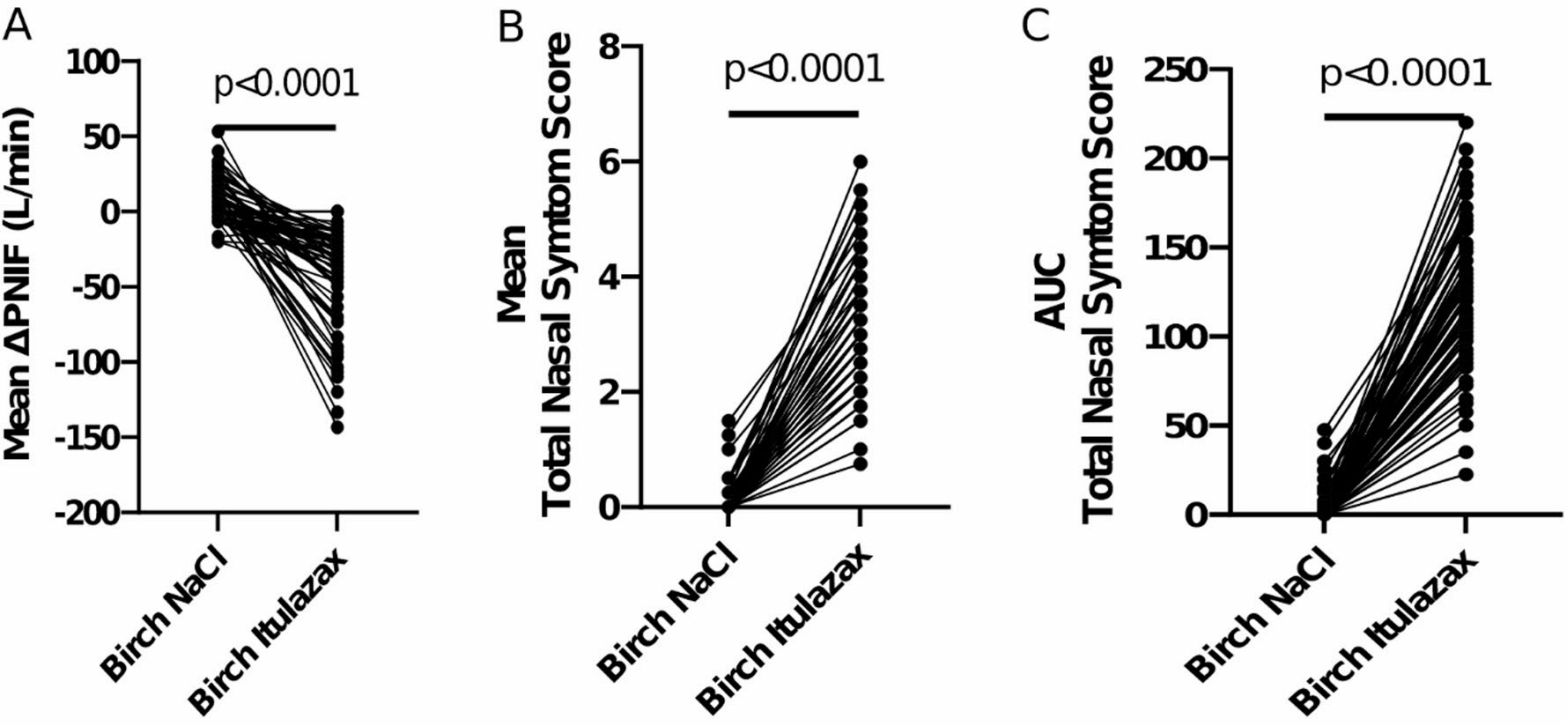
Individual values with paired t-tests of (**A**) mean ΔPNIF, (**B**) mean TNSS, (**C**) TNSS AUC.

A paired t-test comparing provocations with 0.9% saline solution and ITULAZAX in the birch allergy group showed a significant difference (*p* < 0.0001) for the mean TNSS, mean PNIF, and the area under the curve (AUC) for TNSS 0–30 min (Fig. 3).

No adverse events occurred throughout the challenges, and no patient reported prolonged rhinitis or conjunctivitis symptoms following the provocation.

The trial was monitored by an independent monitor. This is to ensure that the trial was carried out according to the protocol and that data are reliable and robust and are collected, documented, and reported according to ICH-GCP and applicable ethical and regulatory requirements. There was a single protocol deviation: 69 participants were recruited for the birch allergy group instead of the planned 70 due to time constraints. Extending the study beyond December 2024 would have required transferring the study protocol from the EudraCT database to the CTIS, which would have demanded significant resources.

## Discussion

NAC is an essential diagnostic tool for allergic rhinitis and a valuable method in clinical research. The discontinuation of Aquagen SQ pollen extract, previously used in Sweden and other European countries, has created a need to explore alternative allergen sources for NAC. Our study investigates the use of dissolved ITULAZAX tablets as a potential substitute for nasal birch pollen allergy challenge. This approach is in line with the work of Eifan et al.^13^ who evaluated clinical responses to timothy grass pollen allergy using both traditional aqueous extracts and newly developed lyophilizate tablets.

Both studies utilized a cross-over trial design, with Eifan et al. focusing on timothy grass pollen and our study on birch pollen. In terms of clinical efficacy, both studies achieved high sensitivity and specificity in detecting allergic reactions using dissolved tablets for NACs. Eifan et al. reported no significant differences in TNSS between the aqueous extracts and tablet forms, aligning with our findings of 96% sensitivity and 95% specificity with ITULAZAX. These results suggest that tablet forms can reliably reproduce clinical symptoms necessary for a valid NAC, confirming their potential as effective substitutes for the discontinued aqueous extracts.

The use of dissolved tablets simplifies the allergen preparation process, potentially enhancing patient adherence and motivation for SLIT (Sublingual Immunotherapy) due to the familiarity with the allergen substance. In alignment with the EAACI Position Paper on the standardization of nasal allergen challenges^4,6^, our study adopted a standardized allergen source and controlled challenge protocol, providing important evidence in support of standardized NAC approaches.

A single potentially false-negative result in the healthy control group highlights that PNIF measurements may vary due to nonspecific nasal reactivity, anatomical differences, or procedural factors. This underscores the importance of interpreting PNIF in conjunction with patient-reported symptoms to ensure an accurate and comprehensive assessment of nasal responses.

While our study was a pilot investigation without pre-defined power calculations, the promising results, along with the similarities to the GRAZAX study, support the readiness of this method for immediate implementation in clinical practice and research. The favorable outcomes observed with ITULAZAX tablets emphasize their significant utility, offering a highly effective and standardized approach to NAC that aligns with current guidelines. This approach not only addresses the gap left by the discontinuation of other allergen extracts but also holds promise for broader application across various allergenic sources.

## Supplementary Information

Below is the link to the electronic supplementary material.

Supplementary Material 1

Supplementary Material 2

## Data Availability

The datasets generated and analyzed during the current study are available from the corresponding author upon request.
