# Supplementary material for: Nasal allergen challenge with dissolved birch tree pollen tablets
Source: Sci Rep. 2025 Nov 10;15:39355. doi: 10.1038/s41598-025-27356-4 (PMC12603229; doi:10.1038/s41598-025-27356-4)
Supplement: Supplementary file 1 — Supplementary Material 1 [file 41598_2025_27356_MOESM1_ESM.docx]

**Appendix 1:** Inclusion/exclusion criteria

Inclusion criteria

• Written informed consent obtained before any trial related assessments are performed

• Age 18–65 years at the time of consent

• A female participant of child-bearing potential must have a negative pregnancy test and be willing to practice appropriate highly effective birth control methods as listed below until end of Visit 2. The definition of a female patient of childbearing potential is a nonmenopausal female who has not had a hysterectomy, bilateral oophorectomy, or medically documented ovarian failure.

Highly effective birth control methods:

• combined (estrogen and progestogen containing) hormonal contraception associated with inhibition of ovulation:

o oral

o intravaginal

o transdermal

• progestogen-only hormonal contraception associated with inhibition of ovulation:

o oral

o injectable

o implantable

• intrauterine device (IUD)

• intrauterine hormone-releasing system (IUS)

• bilateral tubal occlusion

• vasectomised partner

• sexual abstinence

• Birch pollen allergy group: A documented clinically relevant history of moderate to severe persistent birch pollen allergy symptoms since at least 2 years with troublesome symptoms despite the use of symptomatic medication with or without asthma; positive SPT response (wheal diameter ≥3 mm) to birch pollen (birch) and positive specific IgE to birch (≥ IgE CAP class 2; ≥0.70 kU/L) at screening

• Non-allergic control group: A documented negative clinical history for allergic rhinitis, negative SPT response (wheal diameter <3 mm) and negative sIgE (IgE CAP class 0) to birch pollen (birch), grasses, house dust mite, cat, horse, dog, ragweed, mugwort and mould (Alternaria alternata).

• Participant must be willing and able to comply with the trial protocol

Exclusion criteria

• A participant in the birch pollen allergy group with rhinitis and/or conjunctivitis caused by animal hair and dander to which the subject is regularly exposed is not eligible for the trial. In terms of seasonal and perennial allergens like moulds etc. the subject can be sensitised to these allergens but is not eligible for the trial if the subject has symptoms induced by these allergens during the trial period.

• Participants who have experienced a severe asthma exacerbation within the last 3 months.

• Reduced lung function (in adults: FEV1 < 70% of predicted value after adequate pharmacologic treatment).

• SLIT treatment with birch pollen for more than 1 month within the last 5 years. In addition, any SLIT treatment with birch pollen within the previous 12 months.

• SCIT treatment with birch pollen reaching the maintenance dose within the last 5 years. In addition, any SCIT treatment with birch pollen within the previous 12 months.

• Ongoing treatment with any allergy immunotherapy product

• Any nasal oropharyngeal condition that might mimic birch pollen allergy symptoms (e.g. strong septal deviation, nasal polyps, a history of paranasal sinus surgery or surgery of nasal turbinate's; choanal atresia, hypertrophy of pharyngeal tonsil, naso/oropharyngeal tumours, hypertrophy of palatial tonsils). For exclusion both sided nasal endoscopy including endoscopy of the epipharynx is required.

• Any general condition that might induce birch pollen allergy symptoms (hypothyroidism, pregnancy).

• Any pharmacotherapy that might induce birch pollen symptoms (e.g. treatment with antihypertensive drugs, treatment with cholinergic acting drugs like antidepressants).

• History of any other (allergic) rhinitis symptoms than to birch pollen during the whole study period for subjects in the birch pollen allergy group.

• A relevant history of systemic allergic reaction e.g. anaphylaxis with cardiorespiratory symptoms, generalised urticaria or severe facial angioedema that in the opinion of the investigator may constitute an increased safety concern.

• Any clinically relevant chronic disease incl. malignancy that in the opinion of the investigator would interfere with the trial evaluations or the safety of the subject.

• Active or poorly controlled autoimmune diseases, immune defects, immunodeficiencies, immunosuppression or malignant neoplastic diseases with current disease relevance.

• Immunosuppressive treatment (ATC code L04 or L01) within 3 months prior to the screening visit.

• Treatment with medications with potential impact on NAC (e.g. treatment with anti-IgE or anti IL-5 drugs within 130 days/5 half-lives of the drug (which ever longest) or treatment with antidepressant or antipsychotic medications with antihistaminic effect).

•Current participation in other clinical trials and or use of an investigational drug within 30 days/5 half-lives of the drug, which ever the longest, prior to screening.

• A history of allergy, hypersensitivity or intolerance to the IMP (except birch pollen).

• Smoking / significant history of smoking.

• A history of alcohol or drug abuse.
