# Supplementary material for: Nasal allergen challenge with dissolved birch tree pollen tablets
Source: Sci Rep. 2025 Nov 10;15:39355. doi: 10.1038/s41598-025-27356-4 (PMC12603229; doi:10.1038/s41598-025-27356-4)
Supplement: Supplementary file 2 — Supplementary Material 2 [file 41598_2025_27356_MOESM2_ESM.docx]

**Appendix 2:** Preparation of extracts for NAC ITULAZAX®

Preparation of solution for NAC with Birch lyophilizate tablet (ITULAZAX®)

ITULAZAX® 12 SQ-Bet ≈ Aquagen 600 000 SQ-U

One 12 SQ Bet tablet of purified Betula Verrucosa lyophilizate is placed into a sterile specimen container; 6ml of sterile 0.9% saline is added, dropwise, using a 1ml syringe, onto the tablet, ensuring that the tablets completely dissolves; the solution is then shaken gently to aid mixing. This gives a stock solution with a concentration of 2 SQ-Bet/ml (Corresponding to 100,000 SQ-U/ml Aquagen).

1ml of stock solution is then withdrawn in a sterile 1ml syringe and added to 1ml of saline in a second sterile specimen container and mixed gently. This gives a solution of 1 SQ-Bet/ml (corresponding to 50,000 SQ-U/ml).

New stock solution and serial dilutions is made daily.

Within 1 hour of each challenge beginning 1 SQ-Bet/ml (50,000 SQ-U/ml) solution is added to a spray device, MAD110 Nasal Device, able to provide one 0.1 ml puff/nostril corresponding to a total amount of 0,2 SQ-Bet (10 000 SQ-U) N.B. A CE-marked nasal applicator device will be used (MAD110). The dead-space in the applicator is 0.15 ml. The syringe should be filled with 0.25 ml 1 SQ-Bet/ml solution per nostril (2 x 0.25ml) to administer correct dose
